# Longitudinal record linked analysis of an assertive community treatment programme in a suburban mental health hospital: emergency department presentations, hospital admissions and bed days

**DOI:** 10.1007/s00127-025-02931-2

**Published:** 2025-05-16

**Authors:** Susanne Stanley, Ajay Velayudhan, Amanda Hellewell, Mitul Bhatt, Mohan Isaac

**Affiliations:** 1 Fremantle Hospital and Health Service / Mental Health Unit, South Metropolitan Health Service, Fiona Stanley Fremantle Hospitals Group, Fremantle, Western Australia; 2 University of Western Australia, UWA Medical School/Psychiatry, Perth, Western Australia; 3 University of Western Australia, UWA Medical School/Psychiatry, Harry Perkins Institute of Medical Research, North (QEII), 6 Verdun Street, Perth, 6009 Western Australia; 4 Adult Community Mental Health Service, North Metropolitan Health Service, Perth, Western Australia 6000, Australia

**Keywords:** Assertive community treatment, Mental health, Hospital admission, Emergency department, Mental illness, Record linkage

## Abstract

This study aimed to objectively assess a long-term Assertive Community Treatment (ACT) programme run by a suburban mental health hospital in Western Australia. The study examined the programme by tracking Emergency Department (ED) presentations, hospital admissions and length of hospital stays (bed days) of people with severe mental illness who entered the programme. Between January 2008 - June 2019, 160 clients who attended the hospital had presentation and admission activities assessed at two time periods (1) PRE - the period from each client’s first engagement with the mental health service up to their entering the service’s ACT programme, and (2) DURING– which is the time that each client spent engaged in that ACT programme. No difference was found between ED presentations before the ACT programme as compared to during the ACT programme. Voluntary mental health hospital admissions were significantly lower during the programme than before the programme, but no difference was found for involuntary mental health hospital admissions. Both voluntary and involuntary hospital stays, however, showed a significant reduction in bed days for clients during their time in the ACT programme. This data shows the continued use of the ACT programme at suburban mental health services to be beneficial. While the number of ED presentations and involuntary admissions remained the same (although for different reasons), the reduction in voluntary hospital admissions and hospital bed days suggests that the increased provision of outpatient and home care through ACT is still working to support clients in the community keeping them out of more restrictive hospital settings.

## Introduction

Assertive Community Treatment (ACT) is an intensive specialist programme used to assist people with severe mental health issues [1]. Developed in the 1970’s in Wisconsin, USA, the programme centres upon people who tend to consistently disengage from mental health services, resulting in frequent relapse and hospital readmission [2]. The programme can be utilised by mental health hospitals or community services, providing an intensive home-based treatment for people with severe mental illness, avoiding costly and invasive institutionalised treatments, and assisting clients to become more independent and reintegrate within the community as they recover.

Core principles of the programme emphasise a multidisciplinary team with assertive outreach to engage with clients, taking a holistic approach with training and support to assist clients to reintegrate back into the community, using low staff-client ratios, and offering long-term and continuous care [3]. Success over the years has been seen in countries such as America, Australia and Canada who have shown reductions in many areas - for example, inpatient hospital admissions [1, 4–6]. However, this has not been replicated in the UK (see studies by Killaspy et al.) [7, 8]. Rosen and his colleagues suggest that reasons may have to do with a lack of fidelity to key components of the programme [1]. For example, some services do not offer the extended hours of operation, or ‘time unlimited’ engagement for clients. Odden et al. [9] assessed the model fidelity of ACT in Norway recently, concluding that while team members were satisfied with the model itself and outcomes for clients, implementing the programme into existing services was challenging.

Modifications to the programme have occurred over the years for many reasons though. Kent and Burns [10] cite issues such as model fidelity and furthered this with examples of where the ACT model is much more integrated into UK community mental health services. That is, intensive case management has existed in the UK for many years with well-established Community Mental Health Services. These similarities between ACT and existing services may also inadvertently affect the outcomes of any comparisons made, resulting in little difference in outcomes if the service is already utilising successful aspects of the model. Sommerfeld et al. [11] highlight the success in purposeful adaptations to the ACT programme over time such as the inclusion of Cognitive-Behavioural Social Skills Training (CBSST) to improve client functioning. Adaptations of ACT may be called for depending upon the context in which it is used.

In their examination of ACT services, early randomised control studies were quick to establish model efficacy [12]. The later Wiley-Exley et al. [13] review found a decrease in the likelihood of emergency department visits and inpatient stays, but no change for primary care issues as compared to ‘treatment as usual’. A systematic literature review conducted by Vanderlip et al. [14] examined the cost-effectiveness of general health interventions within ACT programmes. The authors found that most studies reported no increase in overall medical care costs. They found a decrease in emergency room attendance, but they also saw an increase in primary care outpatient visits. Studies of homeless clients with dual diagnoses of mental illness and substance abuse often show a higher level of contact needed, and clients attend substance abuse treatment more often than participants receiving generic case management services. Yet, there is also an improved level of satisfaction for clients and shortened psychiatric hospital stays [15]. Similarly, clients who were frequently incarcerated also showed better outcomes when compared to clients receiving ‘treatment as usual’ [16]. The authors here found that more contact with ACT for clients tended to result in lower prison time and fewer hospital days.

This paper examines whether, more than 45 years on, ACT is still an effective treatment model through the assessment of Emergency Department (ED) presentations, hospital admissions and length of hospital stays (bed days) of people with severe mental illness in a suburban mental health hospital setting. It is hypothesised that the intensive attention that clients receive from the suburban ACT service team is still an effective means of reduction in client ED presentations, hospital admissions and bed days.

## Methods

### Participants

**Fig. 1:**
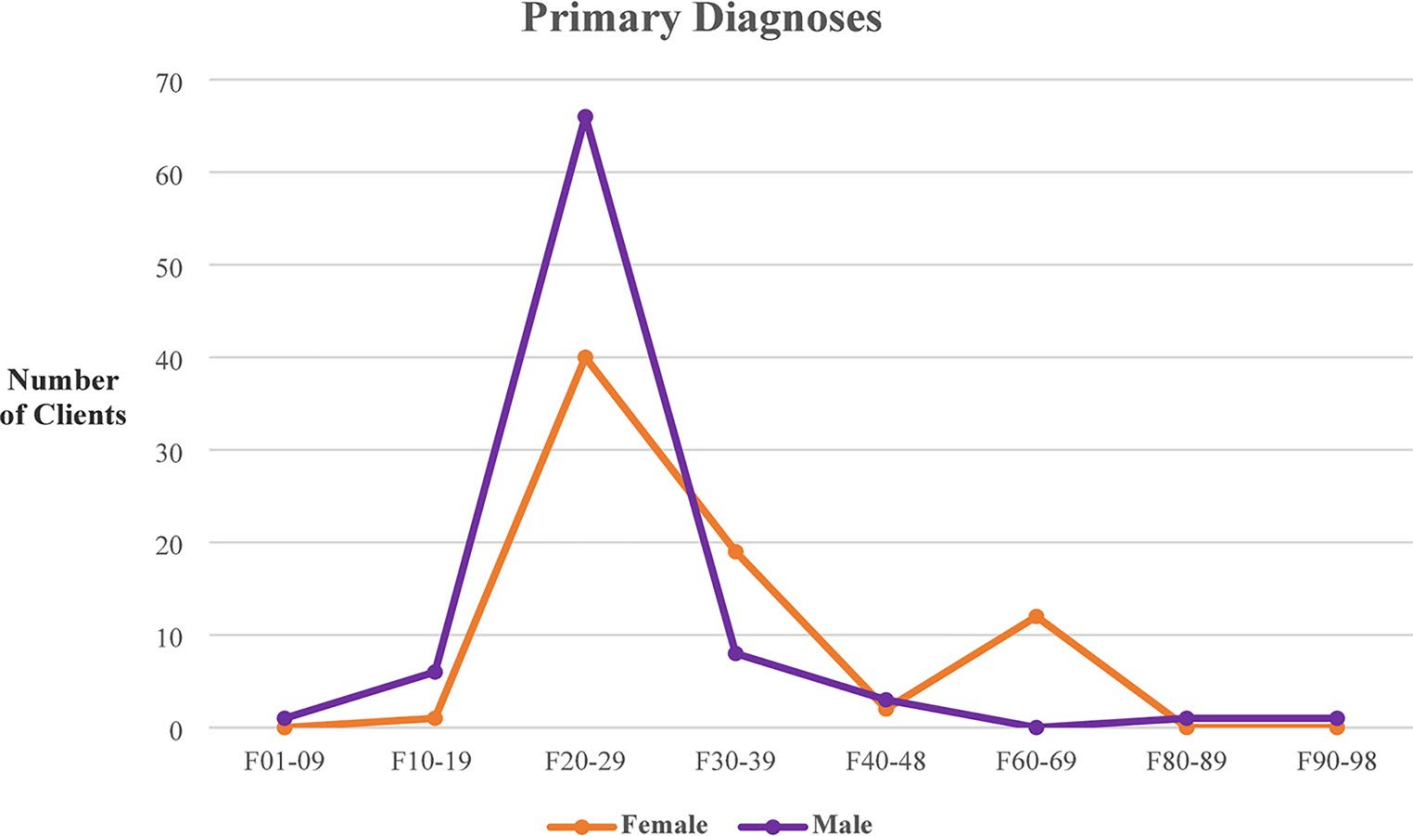
ICD-10 Primary diagnosis of FHMHS ACT clients

This project was approved by the governing ethical review committee of the Fiona Stanley Fremantle Hospital Group– Quality Activity 33,173– and all procedures adhered to the ethical terms, references, and standards of investigation aligning with the principles laid out in the Declaration of Helsinki. The hospital admissions data emanate from a more comprehensive project of ACT clients attending a major hospital in the southern region of the city from January 1992 to June 2019 [17]. This retrospective analysis focused upon the 160 clients who had attended the ACT service beginning January 2008 to June 2019. The narrowing of the time frame was due to the more rigorous data collection procedures that were introduced at this time, and the structural changes within the health service relating to this programme ensured the fidelity of data reported. There were 74 females with a mean age of 45.8yrs (SD13.6), and 86 males with a mean age of 42.9ys (SD11.7). The ICD-10 diagnostic classification criteria (World Health Organisation– WHO) was used to organise primary diagnoses (see Fig. 1.) [18].

### ACT programme admission criteria

To be admitted to the programme, clients had to already be registered with the Fremantle Hospital Mental Health Service (FHMHS), have a severe and enduring mental illness with frequent psychiatric admissions, a history of erratic engagement with mental and/or primary health services and/or medication compliance, have complex co-morbid physical health issues that impact upon their mental wellbeing, or were transitioning from community care to the more concentrated service. Clients who had other issues as well as mental health concerns such as difficulties with illness management in general, substance misuse, accommodation and/or homelessness, social and interpersonal skills, self-care skills, a learning disability, or people who had frequent involvement with the police were also considered for the programme.

The ACT Team (ACTT) at FHMHS is a form of outreach who connects with severely mentally ill adults who, for many reasons, do not engage with the Community service at the hospital. The aim of the team is to reduce the duration and frequency of hospital admissions, educate and encourage medication compliance and stability of mental health symptoms, and assist people with suitable accommodation and financial management. The service highlights early intervention to reduce the incidence of relapse, assisting clients and their families in recognising their specific triggers and enhancing awareness of early signs of relapse.

### Data analysis

Analyses dealt with all occurrences across Western Australia of voluntary and involuntary hospital admissions, mental health Emergency Department presentations, and the length of time clients had spent in hospital (bed days) for each client during the time that the client was engaged/registered as a client with FHMHS. A comparison was made between their engagement with the hospital service (PRE) and their engagement in the ACT programme (DURING). Most clients (75%) had been engaged with FHMHS for approximately two years before they entered the ACT programme. Most clients had been involved with ACT once, but a few clients had engaged with the programme on more than one occasion. Those other occasions were only for very brief periods of time. Clients were not included in the analysis if; (1) they went straight into the ACT programme without first engaging with standard care provisions via FHMHS, (2) their engagements with the ACT programme at FHMHS commenced before 2008, and/or (3) if it was not known whether their hospital admission status was voluntary or involuntary (for a very small percentage of clients, this was not recorded).

Data was collated from the Psychiatric Services On-Line Information System (PSOLIS) in Western Australia, iSoft Clinical Manager (i.CM), the Mental Health Data Collections (MHDC) and the Emergency Department Data Collections (EDDC) via the Department of Health, Western Australia. This enabled the collection of data across catchment areas to follow each client’s trajectory across WA mental health services while they were engaged/registered with FHMHS. Data was then collated and analysed with the IBM SPSS v25 Statistics program.

## Results

### Emergency department presentations

Emergency Department presentations encompassed both physical and mental health issues. Overall, physical health ED presentations increased while clients were engaged with the ACT programme (from a total of 32 presentations PRE ACT to 96 presentations DURING ACT).

**Fig. 2:**
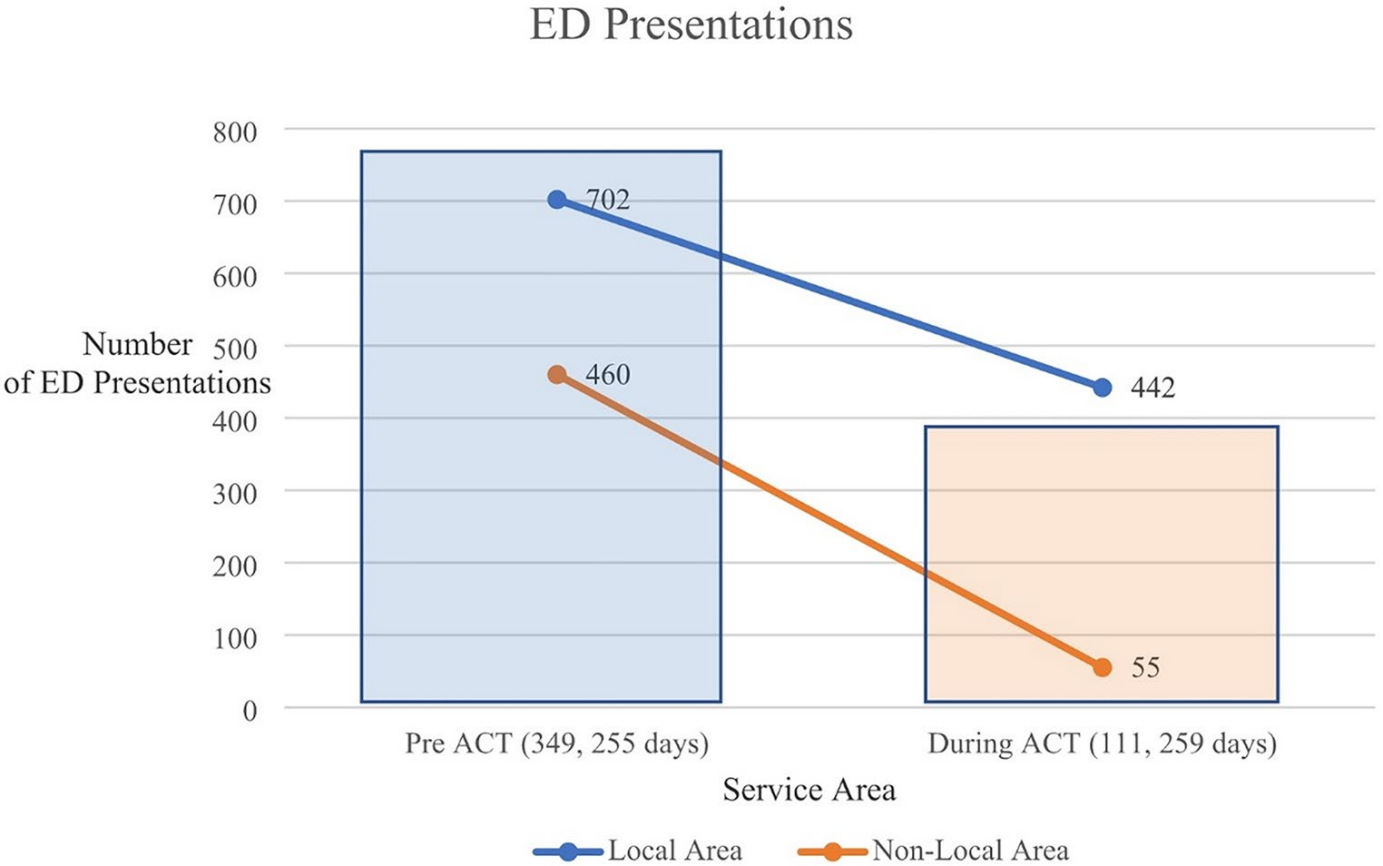
Mental Health Emergency Department presentations before and during the FHMHS ACT programme. * Pre ACT - the time from each client’s first engagement with the mental health service to their entering the service’s ACT programme. ** During ACT - the time that each client spent engaged in the ACT programme at the mental health service

Figure 2 shows that while FHMHS clients were engaged with the ACT programme, if they needed to go to ED for a mental health issue, they were more likely to present in the local catchment area than outside of it. While the number of presentations before they joined the programme were twice as high, the amount of time that clients were engaged with the hospital before they joined the programme was roughly two thirds longer than the amount of time they were engaged in the actual ACT programme. As the two time periods were different, ratio data analysis was used to assess significance.

Ratio data, matched within each time-period (PRE and DURING) for each client was assessed to determine a significant difference in mental health ED presentations. The ratio was set at zero for those instances where the client only had ED presentation/s either before or during the programme. The Shapiro-Wilk test of the 157 pairs of data revealed an abnormal distribution, so a non-parametric Wilcoxon Signed-rank test was conducted. This showed no difference (*p* =.629) between ED presentations PRE and DURING the ACT programme for mental health issues.

### Hospital admissions

Voluntary and involuntary hospital admissions were assessed to determine if the ACT programme assisted in keeping clients out of hospital. The PRE ACT time period showed 1,239 hospital admissions, whereas there were only 466 admissions DURING ACT. Of the 160 clients in the analysis, 145 were hospitalised PRE ACT whereas only 109 experienced hospitalisation DURING the ACT programme.

To assess the significance of whether these admissions were voluntary or involuntary during the two different time periods, a ratio of inpatient hospital admission episodes was calculated for each client and each time period. PRE and DURING ratio data were matched for each client with separate ratios calculated for voluntary and involuntary admissions. In some cases, a client might have had an inpatient episode for only one period of time. In these instances, the ratio for the second time period was set at zero. This resulted in 139 data pairs for voluntary admissions and 153 data pairs for involuntary admissions.

To determine significance, a summary of ratio difference was conducted for voluntary and involuntary hospital admissions (see Table 1.). A much larger variance was observed for voluntary admissions, with the data highly skewed, so a Sign test was conducted testing ratios of PRE and DURING admissions per month. The finding of *p* =.006 (95% CI [-0.03– 0.007]) revealed that DURING the ACT programme, voluntary admissions were significantly lower than they were PRE ACT. Both parametric and non-parametric tests were conducted for involuntary admissions as the assumptions surrounding the test for normality were inconclusive. Non-significance was found in both cases (t-test– *p* =.76, Wilcoxon Signed Rank test– *p* =.26), suggesting no difference between involuntary hospital admissions PRE and DURING the ACT programme.

**Table 1:** Summary of ratio difference for voluntary and involuntary inpatient admissions

Voluntary inpatient admissions					
Minimum	1st Quartile	Median	Mean	3rd Quartile	Maximum
-0.6	-0.077	-0.019	0.027	0.07	2.032
*Involuntary inpatient admissions*					
Minimum	1st Quartile	Median	Mean	3rd Quartile	Maximum
-0.526	-0.069	-0.017	0.003	0.056	0.88

### Bed days

In addition to the number of times a client was admitted to hospital, the time they spent there can also be substantial and is generally calculated as bed days. The summary of ratio difference in Table 2 details the parameters for how long voluntary and involuntary clients stayed in hospital once admitted.

**Table 2:** Summary of ratio difference for voluntary and involuntary inpatient admissions average length of stay/bed days

*Voluntary inpatient length of stay*					
Minimum	1st Quartile	Median	Mean	3rd Quartile	Maximum
-112.0	-18.2	-8	-9.32	2	150
*Involuntary inpatient length of stay*					
Minimum	1st Quartile	Median	Mean	3rd Quartile	Maximum
-212.7	-32	-15.4	-4.06	7.2	419.3

For both types of admission, the range of length of stay was quite broad. For each inpatient episode, the difference in days between the admission date and the discharge date was calculated to find the episode length of stay. As per the hospital admission data analysis, the PRE and DURING data were matched for each client. In cases where a client only had an episode in either one or the other time periods, the value for the missing time period was set to 0.

The data assessment of the distribution for voluntary admission length of stay found a low skew value of 0.47. Therefore, to compare the Pre and During ratio difference a Wilcoxon Signed ranks test was conducted. A significant difference (*p* =.000, 95% CI [-11.435– -5.3]) was found in the average length of stay for voluntary inpatient episodes, showing that the number of bed days reduced DURING the ACT programme. As a high skew (2.89) was found with the involuntary admissions length of stay distribution, a Sign test was used to assess the ratio difference. It was found that the average length of stay for involuntary hospital admissions DURING the ACT programme was significantly lower than the average length of stay PRE the ACT programme (*p* =.000, 95% CI [-20.0– -10.96]).

## Discussion

Assertive Community Treatment provides intensive supports to clients with severe mental health issues with the aim of increasing overall wellbeing and assisting them to continue to live in the community [19]. The admissions and service use data from FHMHS found that clients tended to stay within the same catchment area once they had engaged with the ACT programme. This mirrors the findings of the Dieterich et al. Cochrane systematic review when comparing standard care in the community to a more intensive case management programme (ICM– a combination of ACT and Case Management where ICM has the outreach and community-based components of ACT, but services are provided by a single case manager to a caseload of fewer than 20 clients) [20]. Their overall findings were that clients in the ICM groups were most likely to remain with the service, show an improvement in their general functioning, have accommodation, be employed, and tended to spend less time in hospitals. Over time other modifications and flexibilities to the ACT programme have emerged such as Flexible ACT (FACT), a Dutch version of ACT developed initially to accommodate rural settings allowing easy transition to and from other services [21]. This adaptation has also been shown effective with youth who don’t typically engage with regular mental health services have also been shown to improve their mental health symptomology, and social and personal recovery [22].

Unlike the Wiley-Exley et al. [13] study, no differences were found in mental health ED presentations before as compared to during clients’ time in the ACT programme. Interestingly though, the FHMHS data revealed an increase in the number of primary health presentations to ED during their time in ACT, suggesting that part of the additional consideration given to clients was a focus on physical health issues that needed attention. Primary health care issues are substantial as people with mental health concerns have increased mortality and a higher incidence of chronic disease as compared to the general population, and some clients stop their medications due to debilitating side effects impacting upon their overall health and quality of life [23–25].

A major goal of ACT is to move away from more restrictive treatments such as hospitalisation. The ACT clients in Denmark showed a reduction in psychiatric hospitalisations, and this was most likely to occur over the first two years of engagement with the programme [26]. The assessment of client pathways in the current study found that overall, more clients experienced psychiatric hospitalisations before their engagement with the ACT programme (145 clients) as compared to their time during the programme (109 clients). The investigation found significant differences in the reduction of voluntary hospital admissions whilst in the ACT programme, yet no difference was found for involuntary admissions. This suggests that the more frequent appointments with case managers allowed the picking up of signs that a client was starting to become unwell and to address this, or perhaps gave more time for case managers to assist clients in monitoring their own cues and act on them.

For those who were hospitalised, the current study found that for both voluntary and involuntary admissions, the length of stay was significantly reduced during their time in the ACT programme. This finding warrants further investigation to allow clients to spend less time in hospital and more time at home in the community. For hospital services, there is a likely cost saving through reduced bed days overall, countering the higher costs of the programme via the more concentrated assistance given to clients. The Dieterich et al. [20] Cochrane review and the Thoergersen et al. [27] Denmark study both showed a reduction in bed days due to their ACT programmes. Like the current study, the earlier study also noted that once engaged in the programme, clients tended to stay with the same mental health service. It could be that the low staff-client ratios, a holistic approach to support community reintegration, and using and offering long-term and continuous care allows for a closer, less formal relationship between clients and case managers engendering trust, care and compassion.

### Limitations and strengths

There are some limitations associated with this study. This is a retrospective study with no comparison group. Therefore, no causality can be assumed and ideally, a randomised controlled trial would be needed to infer causality. The small number of clients who went straight into the ACT programme could not be evaluated, and a few clients did not have their hospital status recorded. Data also suggests that to warrant entry to the programme, client mental health had deteriorated for many. In this case, one would expect to see higher numbers of hospitalisations and bed days during ACT due to poorer mental health. More detailed analyses may be needed to further explain findings where finer segregated time period data would highlight individual differences rather than averages.

More broadly, there are limitations in the comparison of the ACT model across services, as pointed out by Kent and Burns [10]. The authors suggest three reasons for poor findings in countries such as the UK– model fidelity, context, and the control condition or comparisons to ‘standard’ care. It is likely that in addition to clear programme outlines, a clear definition of standard treatment is also needed to be able to compare locations and services. A major strength of the study though, is that the data is longitudinal and covers a longer period of time than most other studies of ACT (11.5 years). Also, the outcomes investigated here are those where data are likely to be accurately recorded (Emergency Department presentations, hospital admissions (both voluntary and involuntary) and hospital bed days).

## Conclusion

Mental health services look toward practices and treatments with demonstrated efficacy and effectiveness for assisting clients. Data presented here takes an important step in examining the outcomes of the ACT programme at one mental health service over a lengthy period of time. The programme shows reductions in voluntary hospitalisations and overall hospital bed days for clients, with no differences in emergency department presentations for mental health but a decided improvement in presentations for physical health issues. The ACT service highlights an increased provision of outpatient care and care at home to keep people out of mental health hospitals. A person-centred approach assists people who experience severe mental health issues and supports their ability to remain in their own communities, in their own homes. Despite changes and cultural shifts in the attitudes and behaviours that have occurred in societies over time, the ACT model is still essential today.

## Data Availability

No datasets were generated or analysed during the current study.

## Abbreviations

ACT: Assertive Community Treatment
ED: Emergency Department
CBSST: Cognitive-Behavioural Social Skills Training
EDDC: Emergency Department Data Collections
FHMHS: Fremantle Hospital Mental Health Service
IBM SPSSv25: International Business Machines Statistical Package for the Social Sciences version 25
ICD-10: International Classification of Diseases 10th Revision
ICM: Intensive Case Management
i.CM: iSoft Clinical Manager
MHDC: Mental Health Data Collections
PSOLIS: Psychiatric Services On-Line Information System
WHO: World Health Organization
